# Antidepressant-Like Effect of Lipid Extract of* Channa striatus* in Postpartum Model of Depression in Rats

**DOI:** 10.1155/2017/1469209

**Published:** 2017-11-29

**Authors:** Mohamed Saleem Abdul Shukkoor, Mohamad Taufik Hidayat Bin Baharuldin, Abdul Manan Mat Jais, Mohamad Aris Mohamad Moklas, Sharida Fakurazi, Rusliza Basir

**Affiliations:** ^1^Department of Human Anatomy, Faculty of Medicine and Health Sciences, Universiti Putra Malaysia (UPM), 43400 Serdang, Selangor, Malaysia; ^2^Department of Biomedical Sciences, Faculty of Medicine and Health Sciences, Universiti Putra Malaysia (UPM), 43400 Serdang, Selangor, Malaysia

## Abstract

Postpartum depression affects 15% of women.* Channa striatus*, a freshwater fish, is consumed in local Malay population as a rejuvenating diet during postpartum period. This study evaluated the antidepressant-like effect of lipid extract of* C. striatus* fillet and its mechanism of action in female Sprague-Dawley rats in postpartum model of depression. The rats were ovariectomized and treated with high dose of progesterone and estradiol benzoate for 23 days to have hormone-simulated pregnancy. The day 24 and afterwards were considered as the postpartum period. During the postpartum period, lipid extract was administered at 125, 250, and 500 mg/kg through intraperitoneal route for 15 days. Fluoxetine (10 mg/kg) was used as the positive control. On postpartum day 15, the animals were tested in forced swimming test (FST) and open field test (OFT) followed by biochemical analysis. Withdrawal of hormone administration during the postpartum period induced depressive-like behavior in FST. Administration of lipid extract reversed that depressive-like behavior at 125, 250, and 500 mg/kg in FST. In OFT, it decreased the exploratory activity. The mechanism of the antidepressant-like effect may be mediated through the decrease in plasma corticosterone, increase in plasma oxytocin, and decrease in nuclear factor-kappa B in prefrontal cortex of rats.

## 1. Introduction

Postpartum depression is considered as a type of depression occurring within 4 weeks after childbirth in females [1]. Around 10–20% of women experience postpartum depression [2]. Postpartum depression is characterized by labile mood with prominent anxiety, irritability, and depressive mood [3]. Postpartum depression is considered as a distinct disorder when compared to major depression, probably due to its presumed unique etiology [3]. In females, pregnancy, delivery, and the postpartum period are uniquely characterized by extremely varying hormonal changes. Plasma levels of estrogen and progesterone increase gradually by the end of third trimester and attain levels approximately 50-fold and 10-fold higher than maximal menstrual cycle levels, respectively. These hormones rapidly drop to early follicular phase levels by days 3 to 7 after delivery [4]. This extremely large fluctuation in estrogen levels might be contributing to the onset of postpartum depression [5]. Chronic gestational stress [6, 7], lower serum polyunsaturated fatty acid levels [8], and a single nucleotide polymorphism in tryptophan hydroxylase 2 gene [9] are found to be associated with increased risk of depression during pregnancy and postpartum. Selective serotonin reuptake inhibitors such as fluoxetine [10] and escitalopram [11] are found to be effective in the treatment of postpartum depression.

Based on the hormonal fluctuation concept [4, 5], an animal model was established in rats to reflect the postpartum depression condition [12–15]. In this model, the female rats are ovariectomized bilaterally and subjected to hormonal treatment (progesterone and estradiol) for 23 days to simulate the natural course of pregnancy of a rat. The days 24–27 are considered as postpartum period and the animals are subjected to forced swimming test and other battery of tests meant to assess the antidepressant-like behavior of rodents. Continued estradiol treatment during the postpartum period in this model of rats reversed the depressive-like behavior [12].

Since breastfeeding takes place during the postpartum period, the safety of any pharmacotherapy should consider the risk/benefit ratio of the treatment and consider safer alternatives when there is a risk to the baby. This is the major limitation in the pharmacotherapy of postpartum depression [16]. Hence, safer and efficacious drugs need to be developed. A recent study from our group indicated the antidepressant-like effect of menhaden fish oil which is rich in *ω*-3 fatty acids in postpartum model of depression [17]. Aqueous and lipid extracts of* Channa striatus*, a freshwater fish naturally occurring in Malaysia, showed significant antidepressant-like effect in our previous experiments [18–20]. Also, the flesh of* C. striatus* is consumed in local Malay population as a rejuvenating diet during postpartum period [21, 22]. Based on these evidences, we hypothesized that* C. striatus* may have antidepressant-like effect against postpartum depression. Hence, this study aimed to evaluate the antidepressant-like effect of* C. striatus* extract in an animal model of postpartum depression and the possible mechanism of action.

## 2. Materials and Methods

### 2.1. Animals

Female Sprague-Dawley rats, approximately 9–11 weeks old, weighing between 150 and 190 g were used. The animals were sourced from Takrif Bistari Enterprise, Seri Kembangan, Selangor, Malaysia. All the animals used in this study were cared for and treated humanely in accordance with the protocols specified by the Institutional Animal Care and Use Committee, UPM, and also with the “Principles of Laboratory Animal Care” (NIH Publication number 85–23, revised in 1985). The animals were housed for 2 weeks under controlled conditions for acclimatization before the experiments. These conditions were as follows: light (12 h light/dark cycle, lights on at 7:00 am), temperature (25 ± 1°C), and free access to food and water. The animals were randomly assigned to different groups for the experiments (6 animals per group). All the experimental protocols were approved by Institutional Animal Care and Use Committee (UPM/FPSK/PADS/BR-UUH/00454), UPM.

### 2.2. Ovariectomy

The bilateral ovariectomy was performed as per previously published procedure [13, 15, 17]. The animals were anesthetized by using intramuscular injections of ketamine 80 mg/kg and xylazine 10 mg/kg [17]. The lateral surface of the animals was shaved by using shaving blades. Approximately 2 cm length incision was made on the lateral surface and the skin was opened. The inner muscle was pulled by using bent sharp forceps and a perpendicular cut was made, thus opening abdominal cavity. The oviduct was pulled up by using blunt forceps and clipping forceps was used to clip the oviduct below the ovary. Two knots were made by using the suture catgut (number 2). The ovary (red colored small dotted area) was cut and the forceps clip was released. The oviduct was placed inside the abdominal cavity by using blunt forceps. The inner muscle was sutured by using catgut number 2. The outer skin was sutured in the same manner. Povidone iodine solution (5%) was sprayed on the wounds. The animals were allowed to recover for one week from the surgery and the hormonal injections were started [12, 13, 15, 17]. One group of animals were done with sham surgery and served as sham control.

### 2.3. Hormone-Simulated Pregnancy (HSP)

The hormone-simulated pregnancy (HSP) procedure was followed from previously published protocols [13, 15, 17, 23]. The pregnancy was simulated for 23 days in female rats. During these 23 days, hormone injections were made to simulate the natural variation of hormones. Progesterone and estradiol benzoate (Sigma Aldrich, MO, USA) were dissolved in sesame oil (Sigma Aldrich, MO, USA) and administered to the animals via subcutaneous route as per the schedule given in Table 1. The day 24 and afterwards were considered as the postpartum period.

### 2.4. Preparation of* C. striatus* Extract and Administration

The* Channa striatus* (2 kg) was procured from local wet market in Selangor, Malaysia. The fish were identified by Dr Mohd Shafiq Bin Zakeyuddin, Research Assistant, Department of Environmental Management, Faculty of Environmental Studies, Universiti Putra Malaysia, Malaysia, by following previously published data [24, 25]. A voucher specimen was kept in Human Anatomy Laboratory, Department of Human Anatomy, Faculty of Medicine and Health Sciences, Universiti Putra Malaysia, Malaysia. The fish were killed by a blow to their heads and flesh was separated from bones and fins. The lipid extract was prepared by using the previously published methods [20, 26]. The flesh was minced to a paste in a blender. No water was added in this step. The minced paste (800 g) was mixed with 1600 mL (1 : 2 w/v) of chloroform: methanol (2 : 1 v/v) and stirred continuously for 2 hr and filtered. The residue was extracted again with the same solvent mixture at same conditions for another 2 hr and filtered. The filtrates were combined and allowed to stand for 3 hr for separation of aqueous and organic layer. The two layers were separated and evaporated at 40°C in a Rota vapor (Buchi, Switzerland) to remove the solvents separately. The organic extract was dried using lyophilization. The lyophilized extract of organic layer was 80 mL of oily liquid (63.77 g, 7.97% w/w, weight referring to the wet weight of minced fillet paste). Chemical analysis of lipid extract by gas chromatography revealed the presence of oleic acid (23.38%), palmitic acid (18.55%), caprylic acid (16.73%), linoleic acid (10.82%), stearic acid (7.37%), and docosahexaenoic acid (5.85%) as the major constituents with no detected eicosapentaenoic acid (data not shown). Based on the results of our previous experiments (data not shown), three doses (125, 250, and 500 mg/kg) of lipid extract were selected. The lipid extract was oily in nature at room temperature. An emulsion of lipid extract was prepared in normal saline with 5% Tween 80 (Sigma Aldrich, MO, USA) to produce 125, 250, and 500 mg/kg doses. All the drug solutions were prepared fresh on the day of administration. The lipid extract doses (125, 250, and 500 mg/kg) were administered by intraperitoneal route to separate group of ovariectomized rats that had undergone hormone-simulated pregnancy from day 1 to day 15 of postpartum period. Fluoxetine (Sigma Aldrich, MO, USA) was used as positive control. Fluoxetine was prepared in normal saline containing 5% Tween 80 (Sigma Aldrich, MO, USA) and administered at a dose of 10 mg/kg [27, 28] through intraperitoneal route from day 1 to day 15 of postpartum period to one group of ovariectomized rats that had undergone hormone-simulated pregnancy. One group of ovariectomized animals served as ovariectomized vehicle control group and administered with appropriate vehicle. Another group of ovariectomized animals that had undergone hormone-simulated pregnancy served as hormone-simulated pregnancy (HSP) control group and administered with appropriate vehicle. A group of nonovariectomized animals served as normal control and administered with appropriate vehicle. A group of sham-operated animals served as sham control group and received appropriate vehicle. All drug and vehicle administrations were done at a constant volume of 10 mL/kg.

### 2.5. Forced Swimming Test (FST)

The FST was conducted based on the original method [29] with modifications [13, 17, 20] described in previous studies. The apparatus consisted of a plastic cylinder (25 cm diameter × 50 cm height) filled with 30 cm deep water at 24°C ± 2°C. On postpartum day 14, pretest was conducted for 15 min [12, 17]. The animals were individually allowed to swim for 15 min in the swim tank. After the pretest session, the animals were dried with a towel and placed under a heat lamp for 10 min to avoid hypothermia and returned to their respective cages. The water was changed after a trial with each animal to avoid influence to next animal. On postpartum day 15, same procedure was followed to conduct the test swim session except the duration. The animals were allowed to swim for only 5 min on the test session. The top view of the activity was recorded with a video camera mounted on the ceiling of the behavior test room. The recorded videos were scored by an observer blind to the treatment regimen and duration of immobility was calculated using a stop watch.

### 2.6. Open Field Test (OFT)

The spontaneous locomotor activity was evaluated by following previously described methods [12, 17, 20]. The apparatus consisted of a square box (75 cm × 75 cm with 42 cm height) made up of plexiglass material. Top of the box was not covered and was kept open to see the movement of animal. The floor and all sides of the box were covered with white cardboard material. The cardboard at the floor of the box was drawn with black lines dividing the floor into equal squares of 15 cm × 15 cm.

On postpartum day 15, the animals were transferred to OFT test room and acclimatized for 1 hr. The animals were placed onto the center of the box and allowed to explore the box freely for 5 min. After conducting the test on each animal, the box was cleaned with dry tissue paper first and later with 70% alcohol solution and was allowed to dry in air to avoid the influence of urine and feces of the previous animal on the next animal. The top view of the box was recorded by a video camera mounted on the ceiling. The video recordings were later scored by an observer blind to the treatment regimen for number of squares crossed and number of rearings. After the OFT, the same animals were tested in FST on the postpartum day 15.

### 2.7. Blood and Brain Tissue Collection and Preparation

On postpartum day 15, 30 min after FST, the animals were decapitated by the use of guillotine. The blood and brain tissue sample preparations were carried out as described in the previous publication [20] and as per the recommendations given in the ELISA kit protocols, Cusabio, Hubei Province, People Republic of China. The trunk blood was collected in an EDTA coated tube and plasma was separated by centrifuging at 1000 ×g at 4°C for 15 min. The plasma was aspirated and used immediately for ELISA analysis or stored at −80°C until being analyzed.

The brain was dissected out quickly and carefully and placed on an ice-cold plate. The prefrontal cortex and hippocampus of both sides of the brain were carefully removed [30, 31]. The brain tissues were washed with ice-cold 1x PBS buffer (1 tablet (BR0014G, Oxoid Ltd., UK) dissolved in 100 mL deionized water), weighed, and homogenized in ice-cold 1x PBS solution (100 mg wet tissue in 1 mL 1x PBS) by using Polytron PT-MR 1600 E (Kinematica AG, Switzerland) homogenizer at 1000 rpm for 3 min.

During the homogenization, the Eppendorf tube containing tissue sample was maintained in an ice-cold environment. The homogenates were stored at −20°C overnight and thawed. After two freeze/thaw cycles to break the cell membranes, the homogenates were centrifuged for 5 min at 5000 ×g at 2–8°C. The supernatants were collected and used immediately for ELISA analysis and protein determination or stored at −80°C until analysis. For analysis, either the right or left hippocampus or prefrontal cortex was analyzed. The sample consisted of equal number of right and left hemisphere parts to counterbalance the lateral effects of brain. This procedure was adapted in protein assay as well as in biochemical assays.

### 2.8. Protein Determination

The protein determination was carried out to standardize the expression of results of marker proteins per g of wet brain tissue as described in the previous publication [20]. Protein concentration was determined in hippocampus and prefrontal cortex tissues of rats by using a protein assay kit from Bio-Rad, CA, USA, with dye reagent concentrate (catalog number 500-0006) and bovine serum albumin as standard (Catalog number 500-0002, Bio-Rad). Microassay standard procedure was used. The principle of protein assay is based on the Bradford's method [32]. The homogenates from brain tissue were used for protein analysis. A calibration curve was constructed and the unknown concentrations were intrapolated and expressed as mg/g of wet brain tissue. Each standard and sample were analyzed in triplicate and mean ± SEM were calculated and used for analysis.

### 2.9. Biochemical Analysis

The plasma was analyzed for corticosterone and oxytocin levels as described in the previous publication [20] by using ELISA kits as per the manufacturer's instructions (Cusabio, Hubei Province, People Republic of China). The homogenates from hippocampus and prefrontal cortex were analyzed for serotonin, dopamine, noradrenaline, interleukin-6 (IL-6), nuclear factor-kappa B (NF*κ*B), and brain-derived neurotrophic factor (BDNF) as described in the previous publication [20] by using separate ELISA kits as per the manufacturer's instructions (Cusabio, Hubei Province, People Republic of China). Briefly, a calibration curve was constructed by using the given standards. Each standard and sample were analyzed in triplicate and mean ± SEM were calculated and used for analysis. The unknown sample concentrations were intrapolated from the standard curve and expressed with respect to per gram of protein for IL-6, NF*κ*B, and BDNF.

### 2.10. Statistical Analysis

All the results were expressed as mean ± SEM. The data were analyzed by one-way ANOVA followed by Tukey's multiple comparison test as the post hoc test. All analyses were performed using the software GraphPad Prism version 6.00 for Windows, GraphPad Software, San Diego, California, USA, https://www.graphpad.com/. Effects were considered significant at *p* < 0.05.

## 3. Results

### 3.1. Forced Swimming Test (FST)

**Figure 1:**
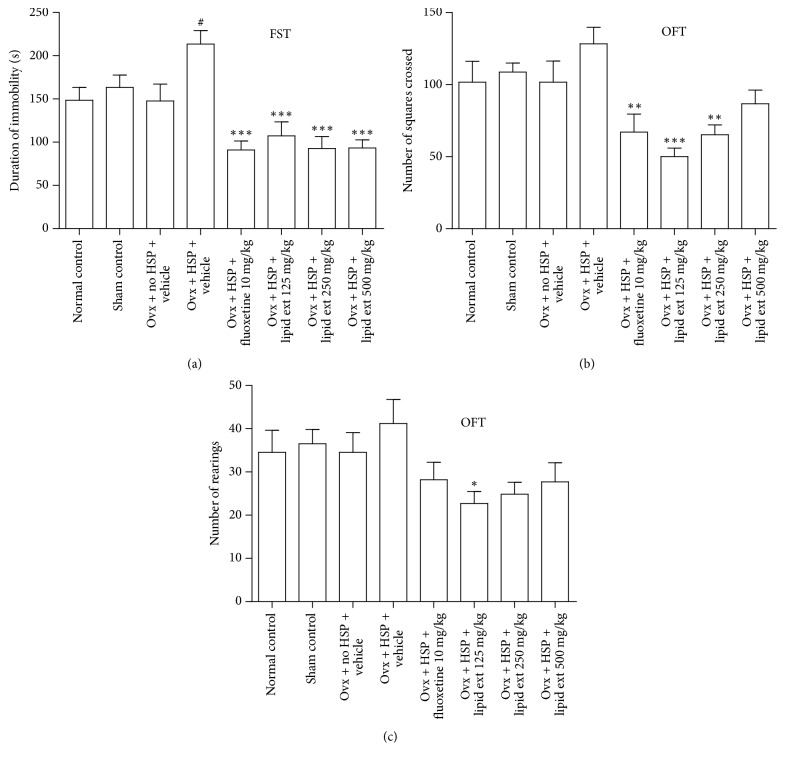
(a) Effect of lipid extract of* C. striatus* fillets and fluoxetine in female rats subjected to postpartum model of depression in forced swimming test (FST). (b) and (c) Effect of lipid extract of* C. striatus* fillets and fluoxetine in female rats subjected to postpartum model of depression in open field test (OFT). Data represent mean ± SEM (*n* = 6). ^#^*p* < 0.05 when compared with Ovx + No HSP + Vehicle group; ^*∗*^*p* < 0.05, ^*∗∗*^*p* < 0.01, and ^*∗∗∗*^*p* < 0.001 when compared with Ovx + HSP + Vehicle group, one-way ANOVA followed by Tukey's multiple comparison test.

In FST, one-way ANOVA indicated significant difference between the treated groups (*F* = 9.102; *df* = 7, 40; *p* < 0.001). With further post hoc analysis, the HSP control group showed significant increase in the duration of immobility (*p* < 0.05) when compared with Ovx + No HSP + Vehicle control group (Figure 1(a)). The lipid extract produced significant decrease in duration of immobility (*p* < 0.001) at 125, 250, and 500 mg/kg doses when compared to HSP control group (Figure 1(a)). The positive control fluoxetine showed significant decrease in immobility (*p* < 0.001) when compared to HSP control group (Figure 1(a)).

### 3.2. Open Field Test (OFT)

In OFT, one-way ANOVA indicated significant difference between the treated groups in number of squares crossed (*F* = 5.961; *df* = 7, 40; *p* < 0.0001) and in number of rearings (*F* = 2.3; *df* = 7, 40; *p* < 0.05). Further post hoc analysis revealed that the lipid extract significantly (*p* < 0.01) decreased the number of squares crossed at 125 and 250 mg/kg doses when compared to HSP control group (Figure 1(b)). Fluoxetine at 10 mg/kg showed significant decrease in number of squares crossed (*p* < 0.05) when compared to HSP control group (Figure 1(b)). The lipid extract significantly (*p* < 0.05) decreased the number of rearings at 125 mg/kg dose when compared to HSP control group (Figure 1(c)).

### 3.3. Biochemical Analysis

#### 3.3.1. Plasma Corticosterone Level

**Figure 2:**
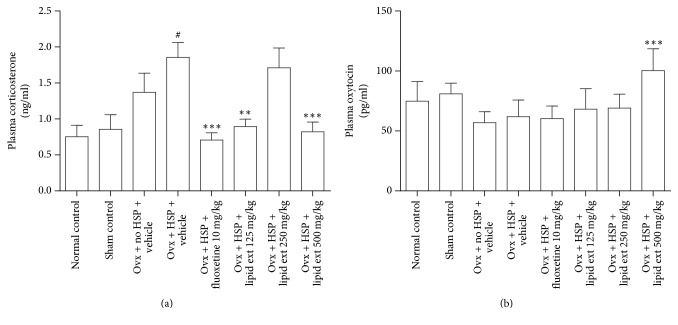
(a) Effect of lipid extract of* C. striatus* fillets and fluoxetine on plasma corticosterone level in female rats subjected to postpartum model of depression. (b) Effect of lipid extract of* C. striatus* fillets and fluoxetine on plasma oxytocin level in female rats subjected to postpartum model of depression. ^#^*p* < 0.05 when compared with sham control and normal control groups; ^*∗∗*^*p* < 0.01 and ^*∗∗∗*^*p* < 0.001 when compared with Ovx + HSP + Vehicle group, one-way ANOVA followed by Tukey's multiple comparison test.

The one-way ANOVA indicated significant difference amongst the treated groups (*F* = 5.699; *df* = 7, 40; *p* < 0.001). Further post hoc analysis indicated that the plasma corticosterone level was significantly (*p* < 0.05) increased in HSP control group when compared with sham control and normal control groups. The lipid extract at 125 and 500 mg/kg doses decreased the plasma corticosterone in a highly significant manner (*p* < 0.01) when compared with the HSP control group (Figure 2(a)). Fluoxetine (10 mg/kg) decreased the plasma corticosterone in a highly significant manner (*p* < 0.001) when compared with the HSP control group (Figure 2(a)).

#### 3.3.2. Plasma Oxytocin Level

The one-way ANOVA test revealed significant difference (*F* = 6.344; *df* = 7, 40; *p* < 0.0001) between all the groups. Further post hoc analysis revealed that the lipid extract at 500 mg/kg dose increased the plasma oxytocin level significantly (*p* < 0.001) (Figure 2(b)).

#### 3.3.3. Brain Serotonin Levels

**Figure 3:**
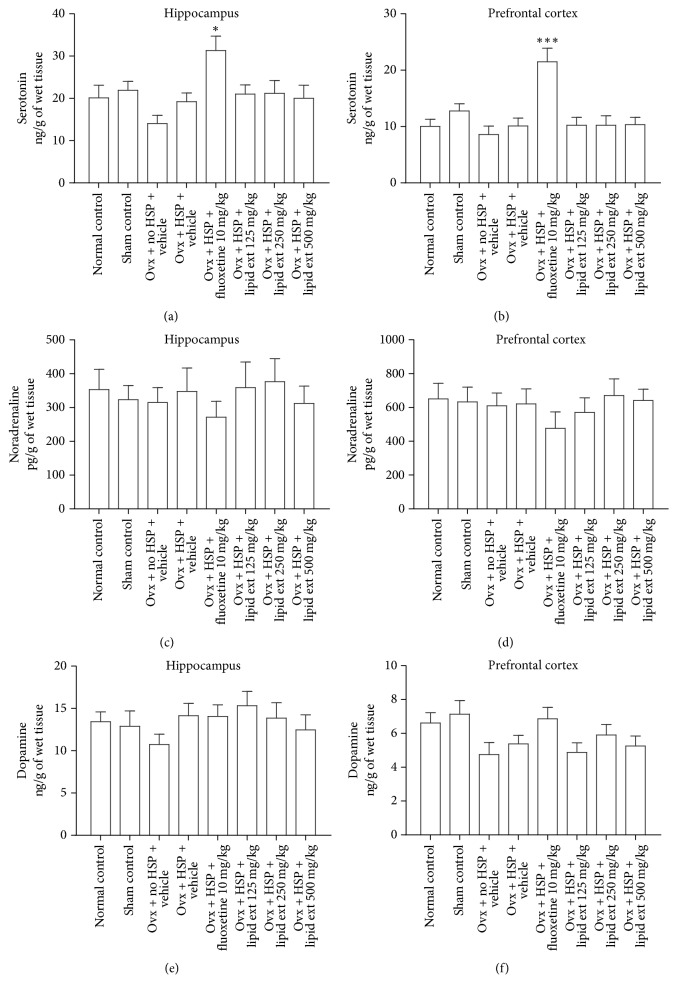
Effect of lipid extract of* C. striatus* fillets and fluoxetine on serotonin level in hippocampus (a), serotonin level in prefrontal cortex (b), noradrenaline level in hippocampus (c), noradrenaline level in prefrontal cortex (d), dopamine level in hippocampus (e), and dopamine level in prefrontal cortex (f) in female rats subjected to postpartum model of depression. Data represent mean ± SEM (*n* = 6). ^*∗*^*p* < 0.05 and ^*∗∗∗*^*p* < 0.001 when compared with Ovx + HSP + Vehicle group, one-way ANOVA followed by Tukey's multiple comparison test.

The one-way ANOVA test revealed significant difference (*F* = 3.254; *df* = 7, 40; *p* < 0.01) in hippocampal serotonin levels and significant difference (*F* = 6.898; *df* = 7, 40; *p* < 0.0001) in prefrontal cortex serotonin levels between the treated groups. Further post hoc analysis indicated that the lipid extract had no significant effect on serotonin level in hippocampus (Figure 3(a)) or in prefrontal cortex (Figure 3(b)) when compared with the ovariectomized HSP control group. Fluoxetine (10 mg/kg) significantly (*p* < 0.05) increased the serotonin level in hippocampus (Figure 3(a)) and very significantly (*p* < 0.001) in prefrontal cortex (Figure 3(b)) when compared to ovariectomized HSP control group.

#### 3.3.4. Brain Noradrenaline Levels

The one-way ANOVA test revealed no significant difference (*F* = 0.3274; *df* = 7, 40; *p* = 0.9369) in hippocampal noradrenaline levels (Figure 3(c)) and no significant difference (*F* = 0.4923; *df* = 7, 40; *p* = 0.8345) in prefrontal cortex noradrenaline levels (Figure 3(d)) between the treated groups.

#### 3.3.5. Brain Dopamine Levels

The one-way ANOVA test revealed no significant difference (*F* = 0.7729; *df* = 7, 40; *p* = 0.6134) in hippocampal dopamine levels (Figure 3(e)) and no significant difference (F = 2.082; *df* = 7, 40; *p* = 0.0681) in prefrontal cortex dopamine levels (Figure 3(f)) between the treated groups.

#### 3.3.6. Brain IL-6 Levels

**Figure 4:**
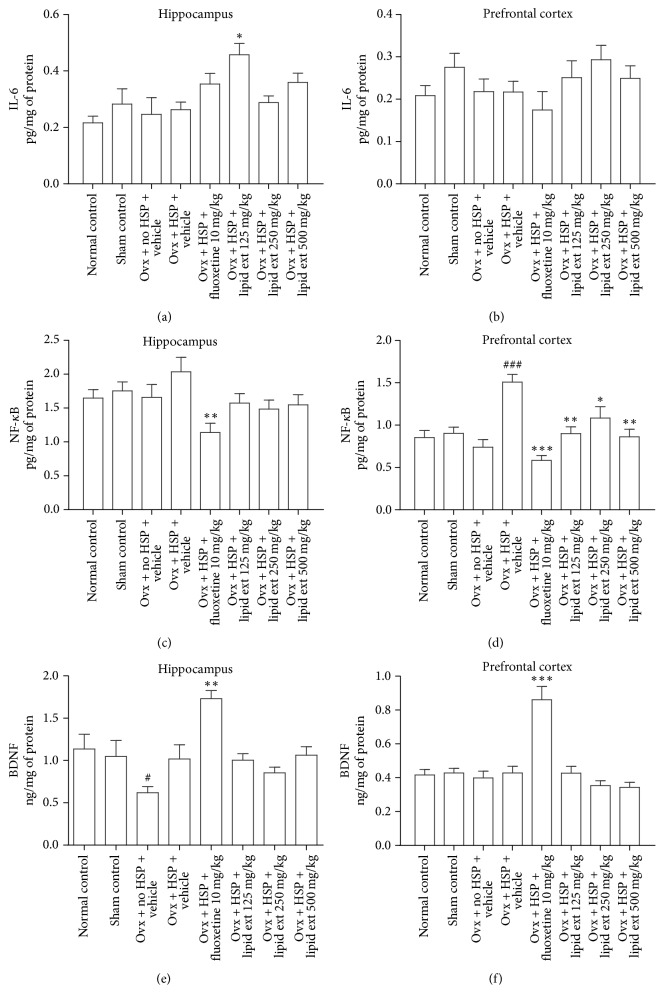
Effect of lipid extract of* C. striatus* fillets and fluoxetine on IL-6 level in hippocampus (a), IL-6 level in prefrontal cortex (b), NF-*κ*B level in hippocampus (c), NF-*κ*B level in prefrontal cortex (d), BDNF level in hippocampus (e), and BDNF level in prefrontal cortex (f) in female rats subjected to postpartum model of depression. Data represent mean ± SEM (*n* = 6). ^#^*p* < 0.05 and ^###^*p* < 0.001 when compared with Ovx + No HSP + Vehicle group; ^*∗*^*p* < 0.05, ^*∗∗*^*p* < 0.01, and ^*∗∗∗*^*p* < 0.001 when compared with Ovx + HSP + Vehicle group, one-way ANOVA followed by Tukey's multiple comparison test.

The one-way ANOVA test revealed significant difference (*F* = 3.886; *df* = 7, 40; *p* < 0.01) in hippocampal IL-6 levels but no significant difference (*F* = 1.366; *df* = 7, 40; *p* = 0.2463) in prefrontal cortex IL-6 levels between the treated groups. Further post hoc analysis indicated that the lipid extract at 125 mg/kg dose produced significant increase in the IL-6 level in the hippocampus (Figure 4(a)) but not in the prefrontal cortex (Figure 4(b)) when compared with the ovariectomized HSP control group. Fluoxetine (10 mg/kg) produced no significant change in IL-6 levels in the hippocampus (Figure 4(a)) and in the prefrontal cortex (Figure 4(b)) when compared with ovariectomized HSP control group.

#### 3.3.7. Brain NF-*κ*B Levels

The one-way ANOVA test revealed significant difference in hippocampal NF-*κ*B levels (*F* = 2.64; *df* = 7, 40; *p* < 0.05) and in prefrontal cortex NF-*κ*B levels (*F* = 9.351; *df* = 7, 40; *p* < 0.0001) between the treated groups. In further post hoc test, the ovariectomized HSP control group showed very highly significant (*p* < 0.001) increase in NF-*κ*B level in prefrontal cortex (Figure 4(d)) but not in hippocampus (Figure 4(c)) when compared with ovariectomized no HSP control group. The lipid extract had no significant effect on the hippocampus (Figure 4(c)) NF-*κ*B level but it significantly decreased (*p* < 0.05) NF-*κ*B level in the prefrontal cortex at all doses (Figure 4(d)) when compared with ovariectomized HSP control group. Fluoxetine (10 mg/kg) decreased NF-*κ*B levels significantly (*p* < 0.01) in hippocampus (Figure 4(c)) and prefrontal cortex (Figure 4(d)) when compared with ovariectomized HSP control group.

#### 3.3.8. Brain BDNF Levels

The one-way ANOVA test revealed significant difference (*F* = 6.161; *df* = 7, 40; *p* < 0.0001) in hippocampal BDNF levels and significant difference (*F* = 15.31; *df* = 7, 40; *p* < 0.0001) in prefrontal cortex BDNF levels between the treated groups. In further post hoc test, the ovariectomized no HSP control group showed significant (*p* < 0.05) decline in hippocampal BDNF level when compared to sham control group (Figure 4(e)). The ovariectomized HSP control group showed slight increase in the BDNF level in hippocampus (Figure 4(e)) but not in prefrontal cortex (Figure 4(f)) when compared with ovariectomized no HSP control group. The lipid extract showed no significant effect on BDNF level in hippocampus (Figure 4(e)) and prefrontal cortex (Figure 4(f)) when compared with ovariectomized HSP control group. Fluoxetine (10 mg/kg) increased the BDNF levels very significantly (*p* < 0.001) in both hippocampus (Figure 4(e)) and prefrontal cortex (Figure 4(f)) when compared with ovariectomized HSP control group.

## 4. Discussion

### 4.1. Antidepressant-Like Effect of Lipid Extract

This study evaluated the effect of lipid extract of* C. striatus* in postpartum model of depression in female rats. This model of postpartum depression has been reported in previous studies [12–15]. In this model, hormone-simulated pregnancy was established by external administration of progesterone and estradiol benzoate in ovariectomized adult female rats and the sudden withdrawal of hormone administration was reported to induce depression-like behavior in ovariectomized rats [13–15, 17]. Similar findings were observed in this study in FST. The hormone-simulated pregnancy induced depressive-like behavior in FST. The ovariectomized HSP control group displayed increased duration of immobility in FST, suggesting a depression-like behavior [13–15, 17]. The lipid extract at doses 125, 250, and 500 mg/kg significantly reduced the duration of immobility in FST suggesting an antidepressant-like effect. The dose-response produced by the lipid extract in FST appears to be biphasic. Lower and higher doses produced less antidepressant-like effect while the middle dose produced more antidepressant-like effect suggesting a U-shaped dose-response curve. In previously reported studies, compounds such as alnespirone (S 20499) [33] and Δ^9^-tetrahydrocannabinol [34] exhibited U-shaped dose-response. The possible explanation for U-shaped dose-response could be the activation/inactivation of multiple pathways at different doses [34]. Based on these previous suggestions, our results suggest that the antidepressant-like effect of lipid extract may involve multiple pathways. The positive control drug, fluoxetine, significantly reduced the duration of immobility in FST suggesting an antidepressant-like effect similar to a previous postpartum study [17].

Lower serum polyunsaturated fatty acid levels put the pregnant women with a risk of developing postpartum depression [8]. Low intake or lower tissue levels of polyunsaturated fatty acids, especially docosahexaenoic acid, were found to be associated with postpartum depression [35]. Lower seafood consumption and lower content of DHA in mother's milk are associated with higher rates of postpartum depression [36]. Women with deficiency of *ω*-3 fatty acids were at 6 times increased risk of developing antenatal depression when compared to women with no deficiency [37]. Low *ω*-3 fatty acid index during late pregnancy was associated with higher scores of depression after three months postpartum [38]. A clinical study recommended the *ω*-3 fatty acids in the treatment of depression during pregnancy [39]. A recent study indicated the antidepressant-like effect of menhaden fish oil which is rich in *ω*-3 fatty acids in postpartum model of depression in rats [17]. Increased intake of oleic acid was linked with reduced risk amongst women to develop severe depressed mood [40]. In a recent study, medium and long-chain fatty acid mixture containing around 52% of oleic acid produced significant antidepressant-like effect in mice in FST [41]. Evidence indicates that blood level of docosahexaenoic acid is inversely associated with depressive symptoms in a recent study in Japan [42]. All these previous studies suggest that polyunsaturated fatty acids, specifically the *ω*-3 fatty acids, would have a therapeutic effect in perinatal and postnatal depression in women. The lipid extract was found to contain around 23% of oleic acid, 5% docosahexaenoic acid, and 1% alpha-linolenic acid as per the chemical analysis (data not shown). Therefore, it may be anticipated that these fatty acids might have contributed to the antidepressant-like effect of lipid extract of* C. striatus *in this study. However, it cannot be concluded from this study. Further studies are required to identify the particular compound responsible for the observed antidepressant-like effect.

Agents that increase the locomotor activity in open field test, including psychomotor stimulants, convulsants, and anticholinergics, tend to produce a false positive result in FST [43]. Therefore, locomotor activity was assessed in rats in open field test to rule out any psychomotor stimulant activity [44]. The major difference between the antidepressants and the psychomotor stimulants is that the antidepressants would not cause significant increase in motor activity [45]. In open field test in this study, hormone-simulated pregnancy increased exploratory behavior similar to previously reported findings [13, 15]. At the same time, in this study, fluoxetine and lipid extract decreased the exploratory behavior in open field test, similar to our previous results [18, 20]. This hypolocomotion effect of treated animals in open field test indicated the absence of any psychomotor stimulant activity thereby supporting the antidepressant-like effect of the lipid extract observed in the FST.

Although the pharmacological mechanism by which the lipid extract caused a significant decrease in locomotion in the open field test is not clearly understood from this study, decreased spontaneous locomotor activity suggests a possible sedative effect [46]. David et al. [47] suggests that even if a sedative effect is observed in the open field test, antidepressant-like activity may be perceived in the FST. In previous studies, clonidine [48], imipramine [49], desipramine [47], buspirone, ipsapirone, and gepirone [49] produced significant decrease in immobility time in FST and significant decrease in locomotor activity (number of crossings and number of rearings) at doses similar to those that decreased immobility. Furthermore, fluoxetine, zimeldine, and indalpine significantly reduced immobility time in tail suspension test and significantly reduced locomotor activity at doses similar to those that decreased immobility [50]. Collectively, these data indicate that antidepressants can produce decreased locomotor activity in open field test in rodents. Hence, the decreased immobility time and decreased locomotor activity produced by the lipid extract in this study are similar to the previous findings [47–50]. Collectively, these results suggest that the lipid extract may have significant sedative effect at 125 and 250 mg/kg in postpartum model of depression. Further studies are required to elucidate the mechanism of hypolocomotion produced by the lipid extract in the open field test.

### 4.2. Mechanism of Action of Antidepressant-Like Effect of Lipid Extract

#### 4.2.1. Involvement of Brain Monoamines

The monoamine hypothesis suggests that the deficiency of monoamines such as serotonin, dopamine, and noradrenaline may be associated with major depression [51, 52]. Serotonin is involved in the regulation of mood and behavior and dysfunction of serotonergic system has been associated with major depression [53, 54]. Decreased function of the noradrenergic system has been associated with depression [55, 56]. The involvement of dopaminergic system has been suggested in the pathophysiology of depression [57, 58]. Hence, in this study, the brain level of these monoamines was estimated. In the present study, the lipid extract did not significantly alter the serotonin, dopamine, and noradrenaline levels in the hippocampus and the prefrontal cortex of the treated rats, suggesting nonmonoaminergic mechanisms [59] for the observed antidepressant-like effect of lipid extract of* C. striatus* in postpartum model of depression.

In this study, fluoxetine increased the serotonin level in hippocampus and prefrontal cortex of the treated rats significantly. Also, fluoxetine slightly increased dopamine in prefrontal cortex when compared to HSP control group in this study. However, it was not statistically significant. In a previous study, fluoxetine increased serotonin and dopamine in prefrontal cortex of rats [60]. Hence, our findings with fluoxetine are similar to previously reported finding [60]. However, the exact mechanism of fluoxetine in postpartum model of depression needs to be studied further to confirm our findings.

From the previously reported findings, it was found out that the brain monoamines in female rats differ during late pregnancy to postpartum period [61]. In anterior cerebral cortex and hippocampus, noradrenaline and serotonin levels decreased during late pregnancy and increased in the early postpartum period and levels of their metabolites increased in the postpartum period. Conversely, dopamine levels were increased in late pregnancy and decreased in the early postpartum period in anterior cortex, while its metabolite, dihydroxy phenyl acetic acid was decreased in both late pregnancy and the early postpartum period [61].

A study, conducted two decades ago, found out that there was no change in the brain serotonin metabolism in ovariectomized rats [62]. An another study indicated that, at 2 and 4 weeks after ovariectomy, the turnover rates of dopamine and noradrenaline in hippocampus and frontal cortex did not differ significantly between the sham-operated and ovariectomized rats. However, at 2 weeks after ovariectomy, the serotonin turnover in hippocampus and frontal cortex was significantly lower than that of sham-operated rats [63]. These findings indicate that ovariectomy had significant effect on the brain monoamine levels.

Acute injection of estradiol to ovariectomized rats did not alter dopamine and serotonin levels in frontal cortex of rats. However, it increased the dopamine turnover 30 min later [64]. Another study indicated that treatment of ovariectomized rats with 17*β*-estradiol decreased serotonin, dopamine, and noradrenaline in frontal cortex and hippocampus and increased their turnover [65]. In a recent study, 17*β*-estradiol treatment in ovariectomized aged rats increased the serotonin turnover ratio (increased formation of serotonin metabolite, 5-hydroxy indole acetic acid) in hippocampus [66], suggesting enhanced serotonergic neurotransmission. In another previously reported study, estradiol treatment in ovariectomized rats did not cause a significant difference in hippocampal noradrenaline level when compared to ovariectomized control group [67]. A recent study found out that the estradiol treatment in ovariectomized rats influenced dopaminergic neurotransmission in frontal cortex [68]. Collectively, these data indicate that the effect of estradiol on brain monoamines in ovariectomized rats is mixed and may vary depending on the dose, duration of treatment, and brain region studied [63].

All these previous studies [61–68] used estradiol alone for supplementation after ovariectomy in female rats and primarily studied the effect of estradiol in ovariectomized rats. Our study also employed 17*β*-estradiol. But, our study used high dose of progesterone and 17*β*-estradiol to simulate pregnancy in ovariectomized rats. During the 15 days of postpartum period, no hormone was administered. Hence, the results of our study may not be directly comparable to the results of all these previous studies [61–68]. To the best of our knowledge, our study is the first study that evaluated the brain monoamines in hippocampus and prefrontal cortex of ovariectomized rats subjected to hormone-simulated pregnancy regimen as a postpartum depression animal model. Collectively, these results indicate that the observed antidepressant-like effect of lipid extract in postpartum model of depression in rats might not be mediated through extracellular increase in the serotonin, dopamine, and noradrenaline.

#### 4.2.2. Involvement of Plasma Corticosterone and Oxytocin

Increased plasma corticosterone level is associated with activated stress response due to activation of the hypothalamo-pituitary-adrenal axis. Majority of the patients with major depression was found to have increased plasma corticosterone level [69]. The lipid extract at 125 and 500 mg/kg doses significantly decreased plasma corticosterone levels similar to a previously reported study suggesting an antidepressant-like effect [17]. The effect does not appear to be dose-dependent but appears to be a U-shaped curve with the lower and higher doses producing higher effect while the middle dose is producing no effect when compared with HSP control group. This type of U-shaped pharmacological response indicates the involvement of two opposing biological pathways [70]. In a previous study, eugenol produced a U-shaped pharmacological response in decreasing the corticosterone in rats [71]. Therefore, our results suggest that the lipid extract may act through more than one pathway in HPA axis in reducing corticosterone. Further studies are required to explore these molecular pathways. Furthermore, our results are consistent with the previous reports stating that corticosterone administration to female rats during the postpartum period increased the duration of immobility in the FST [72, 73]. In this study, the plasma corticosterone level was increased in ovariectomized HSP control group when compared with normal control group and sham control group. These results indicate that the antidepressant-like effect of lipid extract may be mediated through decrease of plasma corticosterone suggesting the involvement of hypothalamo-pituitary-adrenal axis. Fluoxetine decreased plasma corticosterone level similar to a previously reported finding [17].

Oxytocin has been linked with depression [74]. Previous studies indicated the antidepressant-like effect of oxytocin in animals [75, 76]. Therefore, plasma level of oxytocin was evaluated in this study. Plasma oxytocin level was increased by lipid extract at 500 mg/kg dose only. The lipid extract increased plasma oxytocin in ovariectomized hormone-simulated postpartum animal model of depression, reiterating the previous findings [75, 76]. However, the involvement of oxytocin in the neurobiology of postpartum depression needs to be evaluated further.

#### 4.2.3. Involvement of BDNF

Previous studies indicated that decrease in BDNF is associated with stress disorders and depression [77, 78]. Therefore, brain level of BDNF was estimated in this study. The lipid extract had no significant effect on BDNF level in the hippocampus and the prefrontal cortex, suggesting that the antidepressant-like effect may not be mediated through BDNF. The ovariectomy produced significant decrease in the BDNF level in the hippocampus of ovariectomized control group when compared to sham control group. The hormonal treatment slightly increased BDNF level in the hippocampus of ovariectomized HSP control group rats. However, it was not statistically significant. Previous studies showed that ovariectomy decreased BDNF level in hippocampus [79–81] but not in the prefrontal cortex [79, 82]. It appears that estrogen has a direct modulatory effect on BDNF expression in hippocampus of rats [80, 83]. A previous report indicates that treatment of ovariectomized rats with 17*β*-estradiol increased BDNF level in hippocampus [66]. Even though our results appear to be similar to that of the previous studies [79–82], our study design is postpartum model of depression and used high doses of progesterone and 17*β*-estradiol followed by a withdrawal period that is different from other previous studies [79–82]. Hence, our results may not be directly comparable to the results of these previous studies [79–82]. Fluoxetine increased the BDNF levels in both hippocampus and prefrontal cortex in this study. In previous studies, fluoxetine increased the BDNF in hippocampus [84, 85] and prefrontal cortex [86, 87] of rodents. However, since our study employed a postpartum model of depression, the effect of fluoxetine on the BDNF expression needs to be studied further in postpartum model of depression.

#### 4.2.4. Involvement of IL-6

IL-6 is a proinflammatory and anti-inflammatory cytokine involved in the homeostasis of various neurological processes [88, 89]. However, IL-6 is usually found to be elevated in plasma of depressed patients [90, 91] especially in treatment-resistant major depression [92]. A previous study indicated that docosahexaenoic acid may increase IL-6 level in human hippocampal progenitor cells treated with IL-1*β* [93]. Similar observation was made in this study. The lipid extract significantly increased IL-6 level in the hippocampus. In the prefrontal cortex, statistically insignificant increase of IL-6 level was observed. Hence, it may be expected that the docosahexaenoic acid present in the lipid extract (data not shown) might have caused the increase in IL-6 level in hippocampus. However, it cannot be concluded from this study without further in vivo studies exploring the level of IL-6 in different brain parts.

Previous studies indicated that ovariectomy increased the IL-6 levels while estrogen replacement decreased the IL-6 level in serum [94] and in uterus tissue [95]. This suggests that estrogen has a suppressor effect on IL-6 level. In our study, no significant changes in IL-6 levels were observed between the ovariectomized control group and ovariectomized HSP control group. This difference might be due to the difference in the study design used. Our study employed postpartum model of depression based on hormone-simulated pregnancy followed by withdrawal of hormone administration. Hence, the results could be different. However, the suppressor effect of estrogens on IL-6 level cannot be ruled out.

Fluoxetine slightly decreased (statistically insignificant) IL-6 level in prefrontal cortex. A previous study indicated that four weeks of fluoxetine treatment given to C57BL/6 mice increased the production of IL-6 in splenocytes [96]. A clinical study indicated that 6 weeks of fluoxetine treatment in humans increased the production of IL-6 in blood [97]. However, a clinical study indicated no change in blood IL-6 levels after fluoxetine or eicosapentaenoic acid treatment for 8 weeks [98]. The effect of fluoxetine treatment on the blood IL-6 level appears to be mixed. A review indicates that different antidepressant drugs may have divergent effects on the IL-6 levels. Some drugs increase the IL-6 level while some drugs decrease the IL-6 levels [99].

Cytokines, especially IL-6 can cross the blood brain barrier in both directions [100, 101] and hence it may be anticipated that the blood levels may correlate well with the brain levels. However, evidence indicates that, within the brain, the IL-6 expression may be region-specific [102]. Therefore, region-specific differences may be expected in IL-6 expression in brain with antidepressant treatment. In a previous study, fluoxetine (20 mg/kg/day, p.o.) treatment for 7 days in mice attenuated the lipopolysaccharide-induced increase in IL-6 level in prefrontal cortex [103]. Likewise, fluoxetine produced a mild increase in IL-6 level in hippocampus and a mild decrease in IL-6 level in the prefrontal cortex in this study, although the results were statistically insignificant.

#### 4.2.5. Involvement of NF-*κ*B

The NF-*κ*B is a critical mediator of inflammatory processes [104] and upregulation of NF-*κ*B activity has been observed in chronic stress [105, 106]. Cytokines such as IL-1*β* activate NF-*κ*B signaling to constitute an inflammatory response [107]. A statistically insignificant increase in NF-*κ*B level was observed in hippocampus of ovariectomized HSP control rats. However, in the same ovariectomized HSP control group, significant increase in NF-*κ*B level in the prefrontal cortex was observed. A similar pattern was observed in the IL-6 level also. These results suggest that the withdrawal of hormone administration precipitated the inflammatory response in the postpartum period.

The lipid extract significantly decreased the NF-*κ*B level in the prefrontal cortex at 125, 250, and 500 mg/kg doses but not in the hippocampus. A recent report indicates that docosahexaenoic acid can reduce NF-*κ*B level while increasing IL-6 in human hippocampal progenitor cells [93]. Similar findings were observed in our study too, suggesting the involvement of docosahexaenoic acid and its distinct modes of action. The lipid extract was found to contain around 5% docosahexaenoic acid (data not shown). Fluoxetine decreased NF-*κ*B level in hippocampus and prefrontal cortex of rats subjected to hormone-simulated pregnancy. Previous studies indicated that fluoxetine inhibited NF-*κ*B [108, 109]. Hence, our findings are similar to the previously reported findings.

Since 17*β*-estradiol and progesterone were administered to simulate the hormone-simulated pregnancy in the postpartum model of depression, their role, in the regulation of all the biochemical parameters analyzed in this study, cannot be ruled out. Many studies indicated the role of ovarian hormones in the regulation of corticosterone [110, 111], oxytocin [112], monoamines [65, 66], IL-6 [94, 95], BDNF [66, 79, 80, 82, 83], and NF-*κ*B [113, 114]. Despite the profound effects of these ovarian hormones mentioned in the literature, our study did not produce the same results as that of those previously reported studies. This difference could be due to the difference in the study design.

In our study, 17*β*-estradiol and progesterone were administered to simulate the hormone-simulated pregnancy in female rats. During this period, the effects of these hormones on the above-mentioned biochemical parameters can be expected. However, further in our study, the hormone administration was withdrawn to induce a simulated postpartum period. During this period of 15 days, no hormone was administered and only drug treatment was carried out. It may be possible that, during this period of 15 days, the effects of these ovarian hormones on the ovariectomized rats might have waned out.

Collectively these data suggest that the antidepressant-like mechanism of action of lipid extract may be mediated through non-dose-dependent decrease in plasma corticosterone, non-dose-dependent increase in plasma oxytocin, and non-dose-dependent decrease in NF-*κ*B in prefrontal cortex. Since lipid extract contains many fatty acids, especially, the anti-inflammatory *ω*-3 fatty acids and oleic acid, the anti-inflammatory property of the lipid extract may be postulated as the underlying principle in the antidepressant mechanism of action of lipid extract of* C. striatus *in postpartum model of depression. Further studies are required to assess the effect of lipid extract on other cytokines and anti-inflammatory markers in various regions of brain and in plasma.

## 5. Conclusion

The lipid extract of* C. striatus* showed significant antidepressant-like effect in postpartum model of depression in female rats. The mechanism of the antidepressant-like effect appears to be multimodal and may be mediated through non-dose-dependent decrease in plasma corticosterone, non-dose-dependent increase in plasma oxytocin, and non-dose-dependent decrease in NF-*κ*B in prefrontal cortex. Further studies are required to explore the effect of lipid extract on other cytokines and identify the bioactive compound in the extract.

## Figures and Tables

**Table 1:** Schedule of hormone administration after ovariectomy.

Hormone	Days 1–16	Days 17–23
Estradiol benzoate	2.5 *μ*g/rat/day	50 *μ*g/rat/day
Progesterone	4 mg/rat/day	—
